# DOES POSTOPERATIVE GABAPENTIN ANALGESIA ADD TO DELIRIUM IN OLDER COLORECTAL SURGERY PATIENTS?

**DOI:** 10.1093/geroni/igad104.3290

**Published:** 2023-12-21

**Authors:** Ashna Rajan, Joao Filipe G Monteiro, Nadia Mujahid, Matthew Vrees, Steven Schechter, Lynn McNicoll, Stefan Gravenstein, Mriganka Singh

**Affiliations:** Brown University, Providence, Rhode Island, United States; Brown University, Providence, Rhode Island, United States; Brown University, Providence, Rhode Island, United States; Brown Surgical Associates, Providence, Rhode Island, United States; Brown Surgical Associates, Providence, Rhode Island, United States; Brown University, Providence, Rhode Island, United States; Brown University, Providence, Rhode Island, United States; Brown University, Providence, Rhode Island, United States

## Abstract

Our health system adopted a frailty adjusted low-(100 mg) or high-dose(300 mg) gabapentin regimen for opioid sparing postoperative analgesia for older colorectal surgery (CRS) patients to hasten return of bowel function and discharge. But, was it also associated with delirium? 1,306 elective CRS patients ≥ 65 years admitted from 4/2015-6/2023 were retrospectively studied, after excluding those receiving critical care, or hospitalized >3 months. Delirium was defined by ICD coding or postoperative antipsychotic use. Delirium rates were compared using multivariate logistic regression between no- (NG), low- (LG) and high- (HG) dose gabapentin, controlling for age, gender, race, Charlson Comorbidity Index (CCI), depression, preoperative gabapentin use and length of hospital stay. Bivariate, chi-square and t-tests were also used for comparison, as appropriate. NG recipients were found to be older (75.8±7.5) than LG (74.3±6.6, p-value=0.0041) or HG (73.4±6.8, p-value=0.0040), but similar by CCI (mean CCI:6.5±3.9) and depression (17.5%). While overall delirium rates were similar between all three groups, preoperative exposure to gabapentin (as a home medication) was associated with a 3-fold increase in delirium in the HG vs LG (OR 2.76 95% CI [1.25-6.08]) as well as HG vs NG (3.02 [1.38-6.59]) groups. Without gabapentin exposure preoperatively, the NG group was 3 times more likely to develop delirium compared to HG (3.09 [1.36-6.98]). Gabapentin may contribute to postoperative delirium but the unexpected greater delirium incidence among the NG compared to LG may reflect unmeasured confounders of greater underlying delirium risk, or a LG delirium sparing effect.

